# (NZW × BXSB) F1 male mice: An unusual, severe and fatal mouse model of lupus erythematosus

**DOI:** 10.3389/fimmu.2022.977698

**Published:** 2022-09-23

**Authors:** Ruqayyah J. Almizraq, Kayluz Frias Boligan, Melika Loriamini, Colin McKerlie, Donald R. Branch

**Affiliations:** ^1^ Centre for Innovation, Canadian Blood Services, Toronto, ON, Canada; ^2^ The Centre for Phenogenomics, The Hospital for Sick Children, Toronto, ON, Canada; ^3^ Department of Medicine, University of Toronto, Toronto, ON, Canada; ^4^ Department of Laboratory Medicine and Pathobiology, University of Toronto, Toronto, ON, Canada

**Keywords:** lupus erythematosus, sle, endogenous mouse model, autoimmune disease, physical and clinical characteristics, autoantibodies

## Abstract

**Background:**

Systemic lupus erythematosus (SLE) is a chronic autoimmune/inflammatory disease. The heterogeneity and complexity of clinical presentation has made it challenging to study or treat this syndrome. The (NZW×BXSB) F1 lupus-prone male mouse model of this disease is potentially useful to study mechanism and treatment modalities, but there is a lack of information about this model’s characterization and disease progression. Therefore, the aim was to examine this lupus model’s physical/clinical disease presentation and its immunological status.

**Materials and methods:**

Clinical and physical status were observed in 8- and 16-week-old male and female (± 1 week) (NZW/LacJ x BXSB/MpJ) F1 mice (n = 8 per group). Young males (8 ± 1 week) without disease and female (16 ± 1 week) mice served as controls. Physical changes, quantitative values of autoantibodies, and blood cell parameters were determined. Necropsy and post-mortem histopathology were also performed.

**Results:**

With aging (≥ 12 weeks), significant increases in severe abdominal distension/swelling, inability to walk, paleness of paws and significant weight increase were observed compared to controls (p < 0.05). The necropsy examination showed abdominal distension associated with serous effusion and histological examination identified severe edema and multi-organ abnormalities (spleen, lymph nodes, and kidney). Significant increases in anti-double-stranded DNA antibody (anti-dsDNA) was seen in old/sick compared to female (p = 0.0002) or young male (p = 0.0036) mice. Old mice developed immune thrombocytopenia compared to female (p = 0.0056) and young male (p = 0.0007) mice. Anti-platelet was detectable in old, sick mice. The mortality rate increased with aging; more than 35% of male mice died during this study between the ages of 13-18 weeks.

**Conclusion:**

We found that the (NZW/LacJ x BXSB/MpJ) F1 male mice spontaneously exhibit, over varying lengths of time, extremely severe and fatal clinical disease symptoms. This model may be too severe to be helpful in investigating SLE and testing potential treatment modalities.

## Introduction

Systemic lupus erythematosus (SLE), commonly referred to as lupus, is a multisystem chronic autoimmune/inflammatory disease. Lupus is characterized by the autoantibody production against self-antigens (e.g. double-stranded DNA and phospholipids), complement activation and immune complex deposition that result in tissue inflammation and multi-organ destruction (1). While the exact etiopathogenesis of SLE is still not entirely clear, it has been revealed that environmental exposures, cellular, hormonal and genetic factors contribute to the development of this disease (2, 3). The heterogeneity of this disease among patients and the complexity of clinical presentation have made it difficult to study or treat this syndrome potential treatment modalities fo (3, 4). Therefore, several animal models/strains, induced and spontaneous, have been made available to better understand and evaluate the various manifestations and potential this complex human disease (4, 5). While there are several differences among these animal models in terms of the autoimmune disease manifestations, such as the severity of the symptoms, sex differences, age of onset, survival rate, and the progress of autoimmunity (6), each animal model or strain can be an invaluable tool to better understand this disease and for defining pathogenic mechanisms.

F1 hybrids of NZW and BXSB mice develop a spontaneous autoimmune syndrome and have been recognized as a model for autoimmune disease resembling that of human lupus patients. It has been indicated that the development of the autoimmune disease in these mice is severely accelerated in males due to the presence of the Yaa gene (Y chromosome-linked autoimmune acceleration/a mutant gene on the Y chromosome) and not due to hormonal factors (7–9). While it is known that the Yaa gene is the sex-specific factor that provokes the earlier onset, severity of the symptoms and acceleration of autoimmune disease in this mouse model, the mechanism of action remains unclear (9, 10). Even though the (NZW×BXSB)F1 lupus-prone male mouse model of lupus disease is potentially helpful for studying mechanism and treatment modalities, there is a lack of information about this model’s characterization and disease progression. Therefore, this study aimed to examine the physical and clinical disease presentation correlating with the severity of lupus-like disease and the immunological status of (NZW x BXSB)F1 lupus mice.

## Materials and methods

### Animals

NZW/LacJ and BXSB/MpJ were purchased from The Jackson Laboratory. (NZW/LacJ x BXSB/MpJ) F1 lupus mice were generated by crossing a NZW/LacJ female to a BXSB/MpJ male mouse. All mice were housed in standard cages under controlled conditions: five animals per cage, a natural light-dark cycle (12 h light–12 h dark), maintained at 22 ± 4°C and fed with a standard diet and water ad libitum. All mice were maintained under specific pathogen-free conditions. All experiments were completed at the University Health Network (UHN) Animal Research Center (ARC) in Toronto (Animal Use Permit (AUP) 1788.26), and all methods were carried out in accordance with relevant guidelines and regulations.

### Physical characteristics and clinical observation

Clinical and physical status were observed in 8- and 16-week-old F1 males and females (± 1 week) NZW x BXSB F1 lupus mice. For all parameters, 24 mice (n = 8 per group) were used, except for the quantitative values of autoantibodies, where we used n = 28 mice (9 females, 8 healthy/young males, 11 old/sick male). Young male (8 ± 1 week) mice without disease and female (16 ± 1 week) mice served as controls. Clinical/Physical changes, quantitative values of autoantibodies, and blood cell parameters were determined. Necropsy and post-mortem histopathology were also performed.

#### Physical characterization

**Figure 1:**
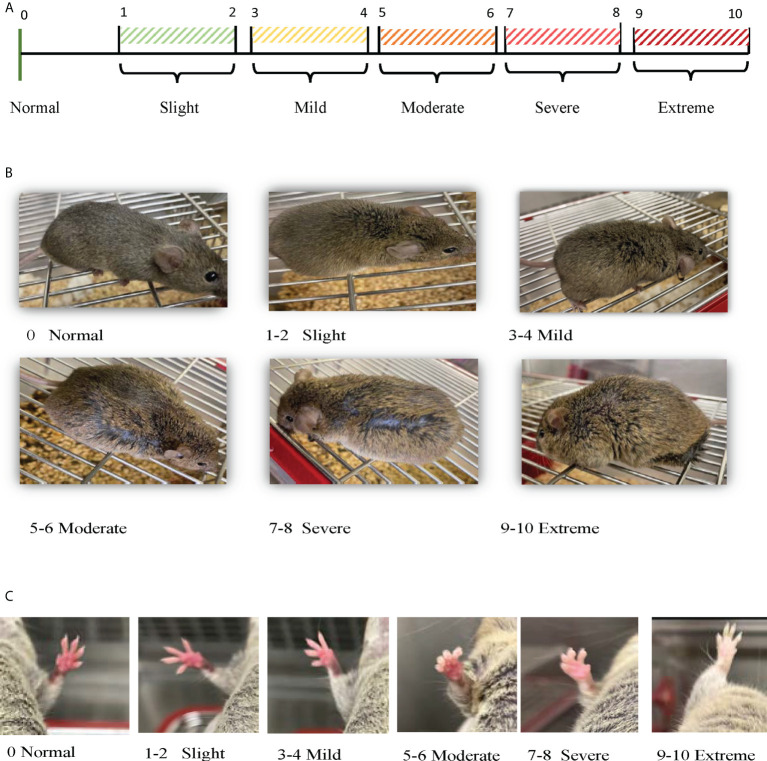
Scoring physical observations. **(A)** Scoring Scale: Numeric rating scale (0 -10-point scale) was used to score the observed changes in the physical status, which include swelling/edema, moving disability and paleness of paws, where 0 indicates no symptom, 1-2 light, 3-4 mild, 5-6 moderate, 7-8 severe, or 9-10 extreme. **(B)** Scoring Swelling: Numeric rating scale (0 -10-point scale) used to score the observed swelling/edema in each mouse during the experimental time (8 – 16 weeks ± 1 week). **(C)** Scoring Paw Skin Coloration: Numeric rating scale (0 -10-point scale) used to score the observed paw skin color changes in each mouse during the experimental time (8 – 16 weeks ± 1 week).

An in-house numeric rating scale (0-10-point scale) for each mouse was used to score the observed changes in the physical status, which include swelling/edema, moving disability and paleness of paws, where 0 indicates no symptom and 10 indicates extremely severe symptoms, as shown in **Figure 1** and **Table 1**. For the total clinical score, the scores of all physical changes were added for a composite score, with a maximum score of 30 per mouse. In addition, the mice were weighed (bodyweight; in grams) and observed for any changes in the clinical and physical status, behavior, and mortality, if any, over a period of 17 weeks. Based on our preliminary study and as this mouse model exhibited sudden unexpected death after 16/17 weeks of age, our experiments for this study were performed within 16 weeks ± one-week.

**Table 1:** Numeric scoring system for physical characteristics of NZW/LacJ x BXSB/MpJ F1 lupus-prone mouse model.

Scoring System and Criteria for physical characteristics of lupus-prone mouse model			
Numeric Scoring	Swelling	Pale paws	Moving disability
**0; Normal**	Normal light slender body with no swelling/edema.	Red/dark pink color for all paws.	Normal activity (running to scape, very strong limb grip/apply force to scape, lid climbing)
**1 -2; Slight**	Slight increase in swelling around the lower body with (1) and without (2) fur/hair loss.	Light changes in the skin color; pink skin color for front (1) and hind (2) paws.	Slight decline in moving ability; walking fast (but not running) when trying to catch the mice, strong limb grip/apply force to scape with (1) and without (2) lid climbing.
**3-4; Mild**	Mild increase in swelling around the lower body and abdominal area with (3) and without (4) fur/hair loss.	Mild changes in the skin color; light pink skin color for front (3) and hind (4) paws.	Mild decline in moving ability; normal walk, limb grip strength declined, apply force to scape with (3) and without (4) lid climbing.
**5-6; Moderate**	Moderate increase in swelling around the lower body and mild swelling around the upper body with (5) and without (6) fur/hair loss.	Moderate changes in the skin color; pale pink skin color for front (5) and hind (6) paws, with or without hypothermia.	Moderate decline in moving ability; slow walk, limb grip strength declined significantly, less force applied to scape with (5) and without (6) lid climbing.
**7-8; Severe**	Severe increase in swelling around the lower and the upper body with (7) and without (8) fur/hair loss.	Severe changes in the skin color; pale skin color for front (7) and hind (8) paws, mostly associated with hypothermia.	Severe moving disability; barley walking and applying force to scape with (7) and without (8) minimum force applied to grip on to a grid.
**9-10; Extreme**	Extreme increase in swelling all over the body including clear swelling around the limbs with (9) and without (10) fur/hair loss. Most of the time under this condition the mouse shows sign of failure to move, eat or drink.	Extreme changes in the skin color; white skin color for front (9) and hind (10) paws, always associated with hypothermia.	Extreme moving disability; not moving at all/dying without (9) or with (10) hypothermia.

The rodent behavioral evaluation can get complicated and test results may differ in the laboratory setting and depend on the people performing the assessment (11). Therefore, all behavioral tests/motor disability tests for this project were assessed by one person during the entire study to maintain consistency and to avoid tech-to-tech variability. To detect differences in motor/moving disability, each mouse was evaluated based on behavioral response from the time that the researcher’s hand reached into the cage to grab the mouse and placing the mouse on top of the wire lid, until the mouse is returned to its cage again. First assessment was observed when reaching into the cage to try catching the mouse. It is normal behavior that the mice run continually around the cage to escape; therefore, any changes in the speed or this behavior were evaluated and recorded as indicated in **Table 1**. Mice were then picked up from the base of the tail and placed on the top of the stainless-steel wire lid to perform the second assessment (grip strength test/limb-hanging test). During grip strength testing, mice placed on the wire lid were then gently pulled back by the base of their tail, ensuring the mouse grips the lid and the mouse is pulled steadily until it loses the grip/lets go. The animal is then placed on the wire lid, which is then inverted and suspended above the home cage; the latency (the time of sustained limb tension to oppose their weight) to when the mouse falls is recorded. This procedure is repeated twice to obtain grip strength measurements and recorded as indicated in **Table 1**. Due to the weakness presented in old sick mice, grip strength and hanging allowed the clear distinction between the groups.

#### Blood collection and testing

Mouse blood was obtained *via* saphenous vein regularly for routine blood tests or *via* cardiac puncture at the end of the study for additional testing.

##### Quantitative values of blood cell parameters

Blood (10 µL) was collected *via* saphenous vein in red top BD microtainer tubes filled with 500 µL of 10% citrate-phosphate-dextrose-adenine (CPDA). Blood tests, including platelet quantification, white blood cells (WBCs) and red blood cells (RBCs) counts, and hemoglobin (Hgb) and hematocrit (Hct) were examined using a Sysmex analyzer (Sysmex pocH-100i hematology analyzer).

##### Quantitation of autoantibodies

Peripheral blood samples were collected *via* cardiac puncture from (NZW x BXSB) F1 mice (old/sick males, young/healthy males and healthy females). Blood samples were allowed to clot for serum isolation, and serum was separated by centrifugation (2000g, for 10 min at 4°C). The isolated serum was used for detecting relevant autoantibodies in SLE and for detecting anti-platelets and anti-red blood cell (RBC) antibodies using flow cytometry.

###### Enzyme-linked immunosorbent assay

Detection of antinuclear antibodies (ANA), anti-dsDNA antibodies, and anti-cardiolipin (CL) antibodies were performed using specific mouse ELISA kits (Ig’s (A+G+M)) from Alpha Diagnostics according to the manufacturer’s instructions.

##### Anti-platelet antibodies

Blood from BALB/c mice was drawn by cardiac puncture in an anticoagulant (K2EDTA) containing tubes for platelet and red blood cell isolation. Platelet-rich plasma was obtained by centrifugation, and platelet concentration was determined using a Z Series Coulter Counter (Beckman Coulter) and adjusted to 10^7^/mL. Carbocyclic PGI2 (Carbacyclin, 1 μg/mL) (Santa Cruz Biotechnology) was added to the platelet-rich plasma to inhibit platelet activation. Platelets were then incubated with NZW x BXSB F1 lupus mice sera (diluted1/8 in FACS buffer, PBS containing 2% FBS, 1mM EDTA) for 1 hour at 4°C, washed and subsequently stained with an Alexa Fluor^®^ 488-AffiniPure Goat Anti-Mouse IgG + IgM (H+L) antibody. Samples were acquired in an SP6800 Spectral Analyzer (Sony Biotechnology) and analyzed with FlowJo V10 (BD Biosciences). Staining with only the secondary antibody was used as isotype control for unspecific antibody binding. Samples were considered positive when the specific staining was at least 5 times higher than the isotype/unspecific binding control.

##### Anti-RBC antibodies

To test for anti-RBC antibodies in (NZW x BXSB) F1 lupus mice sera, RBC from BALB/c mice were isolated, washed and adjusted to 10^6^/tube. BALB/c RBC were incubated with the lupus mice sera (1:8 ratio) for one hour at 4°C, washed and subsequently stained with an Alexa Fluor^®^ 488-AffiniPure Goat Anti-Mouse IgG + IgM (H+L) antibody. Samples were acquired in an SP6800 Spectral Analyzer (Sony Biotechnology) and analyzed with FlowJo V10 (BD Biosciences). TER119 conjugated to APC (Biolegend) was used for specific RBC visualization. Staining with only the secondary antibody was used as isotype control for unspecific antibody binding. Samples were considered positive when the specific staining was at least 5 times higher than the isotype/unspecific binding control.

### Necropsy and histopathology

Necropsy examination were performed by a veterinarian at University Health Network Animal Research Center (Toronto, ON). Organs (heart, lung, kidney, liver, spleen, and lymph nodes) were collected, fixed in 10% neutral buffered formalin and sent to Charles River Laboratories (Charles River Research Animal Diagnostic Services, Wilmington, MA). Sections of paraffin-embedded samples were counterstained with hematoxylin and eosin (H & E) for post-mortem histological analysis.

### Humane endpoints

The endpoint was determined by spontaneous death or elective animal euthanization according to established criteria. For example, sacrificing the animal for necropsy examination and/or collecting samples/tissues post-mortem histopathology, or when signs of severe pain or suffering were shown (e.g. the animal is unable to move, eat or drink, progressive weight loss, or reach an extreme condition in at least 2 physical symptoms as described above).

### Statistical analyses

Statistical analysis was performed using GraphPad Prism, version 8 (GraphPad Software, San Diego, CA; GraphPad Inc). Data were analyzed using analysis of variance with Dunnett posttest for multiple comparisons and to identify significant differences among the groups. Data are presented as scatter dot plot with median line unless otherwise specified. Probability values less than 0.05 were considered statistically significant throughout the study. Significant level (*p < 0.05; **p < 0.01; ***p <0.001, ****p < 0.0001) in comparison to controls have been used.

## Results

### NZW x BXSB F1 old lupus male mice displayed undesirable physical/clinical manifestations of SLE

**Figure 2:**
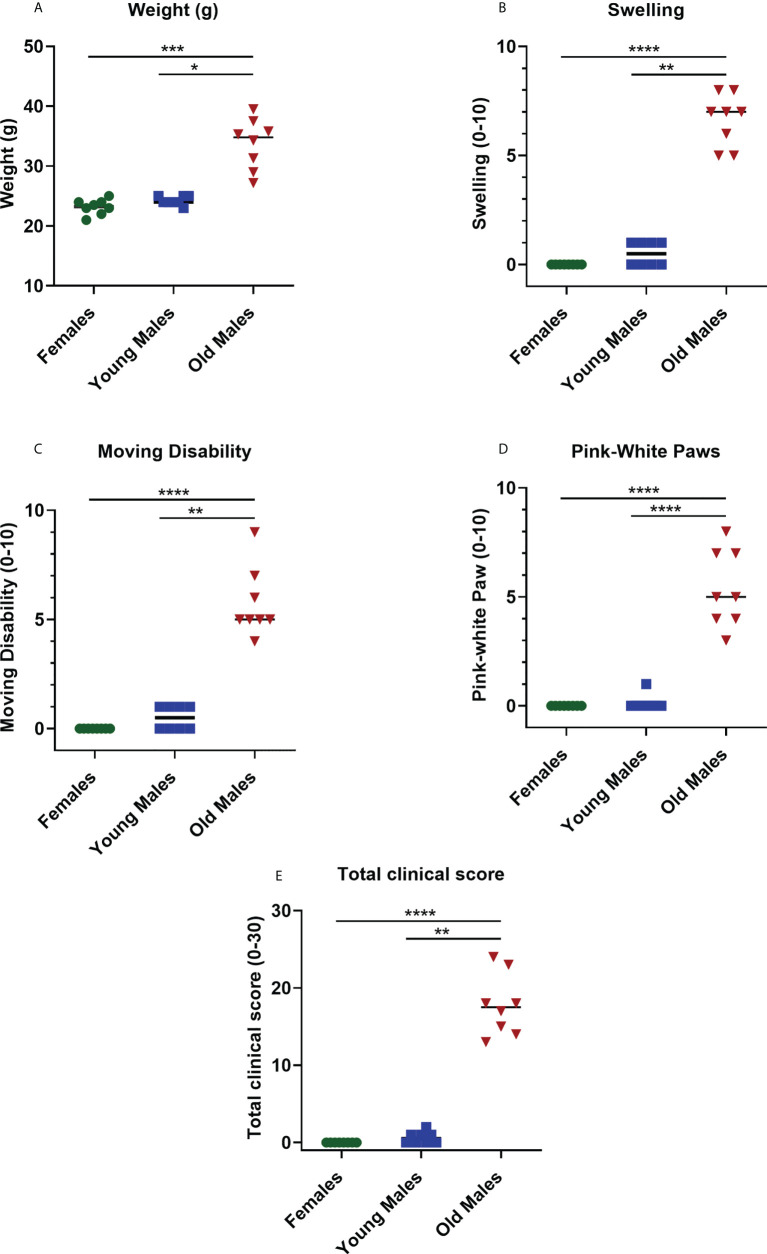
Changes in the physical status of the NZW/LacJ x BXSB/MpJ 9 F1 lupus mice. Body weight **(A)**, swelling/edema **(B)**, moving disability **(C)**, paleness of paws **(D)** and total clinical score **(E)** were measured in females (green circle dots), young/healthy males (blue square) and old sick males (red tringle). Data are presented as scatter dot plot with median line; n= 8 per group. P < 0.05 were regarded as statistically significant. Significant level (*p < 0.05; **p < 0.01; ***p <0.001, ****p < 0.0001) in comparison to controls.

Clinical and physical status were observed in 8- and 16-week-old F1 males and female (± 1 week) of NZW x BXSB F1 mice for any changes as measures of animal well-being. Data showed that the body weight in old/sick lupus mice was significantly higher compared with controls (young males; p = 0.015 and females; p = 0.0001) by more than a 40% increase (**Figure 2A** and **Supplementary Figure 1**). Most of the time, the increase in the body weight was associated with an increase in the swelling, with a body condition scoring (BCS) of 4-5 in most of the sick cases (normal BCS range from 2.5-3). Additionally, moderate to severe abdominal distension/swelling was also observed in all lupus mice in the old male group (≥ 16 weeks), which was statistically significant compared to control groups (young male mice; p = 0.0072 and female mice; p < 0.0001) (**Figure 2B**, **Supplementary Figure 1**). Moreover, monitoring the activity of this mouse model indicated that lupus male mice lose activity (lethargic mice) with aging. Moving disabilities such as inability to walk and difficulties in executing movements as a response to manipulation were associated with the severity of clinical symptoms, duration of illness and age of NZW x BXSB F1 male lupus mice. With aging (≥ 16 weeks), significant increases in movement disability were observed in NZW x BXSB F1 lupus male mice compared to female or young male lupus mice (p < 0.01; **Figure 2C**). While the paw pad color of mice in control groups is pink, old male lupus mice showed abnormal coloration, such as bright pale-white color of their paw pads (**Supplementary Figure 1**). A significant increase in the paleness of the paw pad color was observed with most of the old (≥ 16 weeks) male lupus mice (**Figure 2D**). Overall, as anticipated, the total clinical score was remarkably higher in old sick lupus male mice in comparison to female (p < 0.0001) or young male (p = 0.0074) mice (**Figure 2E**).

### Data of blood cell parameters suggested that lupus male mice developed anemia and thrombocytopenia with aging

**Figure 3:**
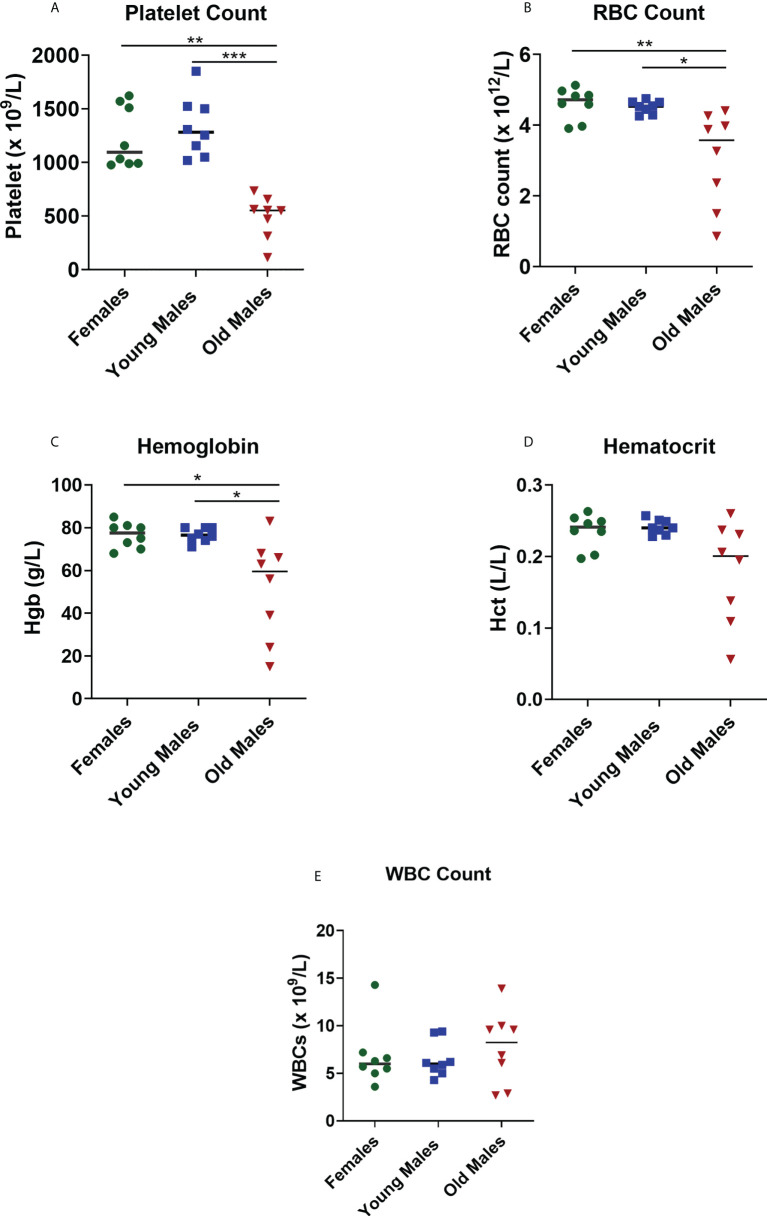
Changes in the blood cell parameters of the NZW/LacJ x BXSB/MpJ 9 F1 lupus mice. Platelets **(A)**, red blood cells **(B)**, hemoglobin **(C)**, hematocrit **(D)**, and WBC **(E)** were measured in female (green circle dots), young/healthy males (blue square) and old sick males (red tringle). Data are presented as as scatter dot plot with median line; n= 8 per group. P < 0.05 were regarded as statistically significant. Significant level (*p < 0.05; **p < 0.01; ***p < 0.001) in comparison to controls.

Complete blood count provides information about health of cells/parameters, all of which were altered by lupus disease. Our findings showed that platelet, RBCs, and Hgb levels were significantly lower (p < 0.05) in old sick males compared to female and young male groups (**Figure 3**). These results suggest that older male mice developed anemia and thrombocytopenia compared to female and young male mice. No significant differences were observed in the number of WBCs between the old sick lupus mice and the control groups (young male mice; p = 0.9653 and female mice; p > 0.9999, **Figure 3E**).

### Detection of autoantibodies in serum of NZW x BXSB F1 old lupus male mice

**Figure 4:**
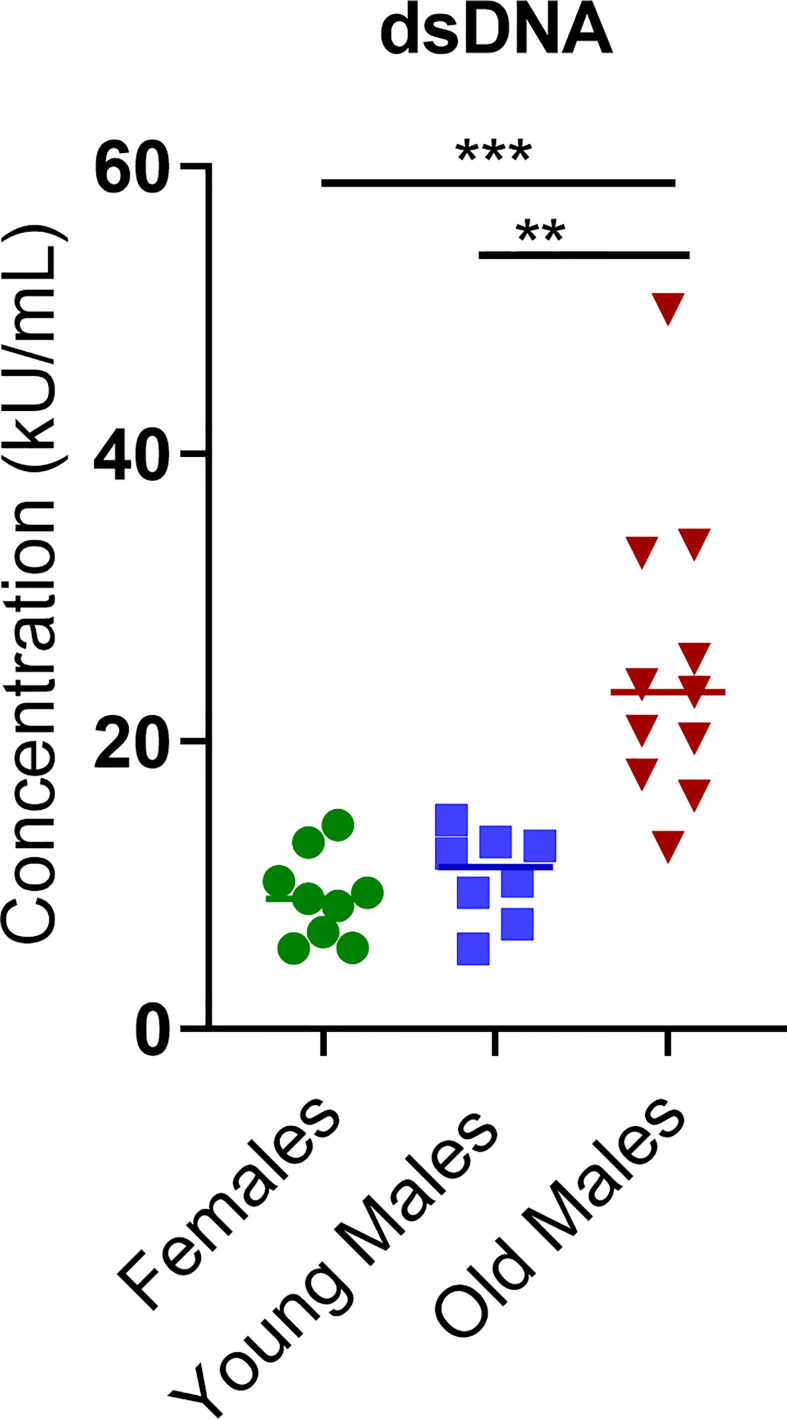
Concentration (kU/mL) of anti-double-stranded DNA antibody (anti-dsDNA) was measured in female (green circle dots, n = 9), young/healthy male (blue square, n = 8) and old sick male (red tringle, n = 11). Data are presented as scatter dot plot with median line. P < 0.05 were considered statistically significant. Significant level (**p < 0.01; ***p < 0.001) in comparison to controls.

A significant increase in the quantitative serum levels of dsDNA antibodies was detected in the sick/old lupus male mice compared to females or young males, consistent with the lupus phenotype in these mice (**Figure 4**). Even though values of the anti-nuclear antigens (ANA) antibodies and anti-Cardiolipin antibodies fell below the detection level of our standard curve (data not shown), the OD values showed that these antibodies were also elevated in the sick males compared to young males and females (**Supplementary Figure 2**). Altogether, these observations confirm the lupus-like phenotype present in this mouse model.

### Histopathological findings revealed multi-organ abnormalities in aged NZW x BXSB F1 lupus male mice

**Figure 5:**
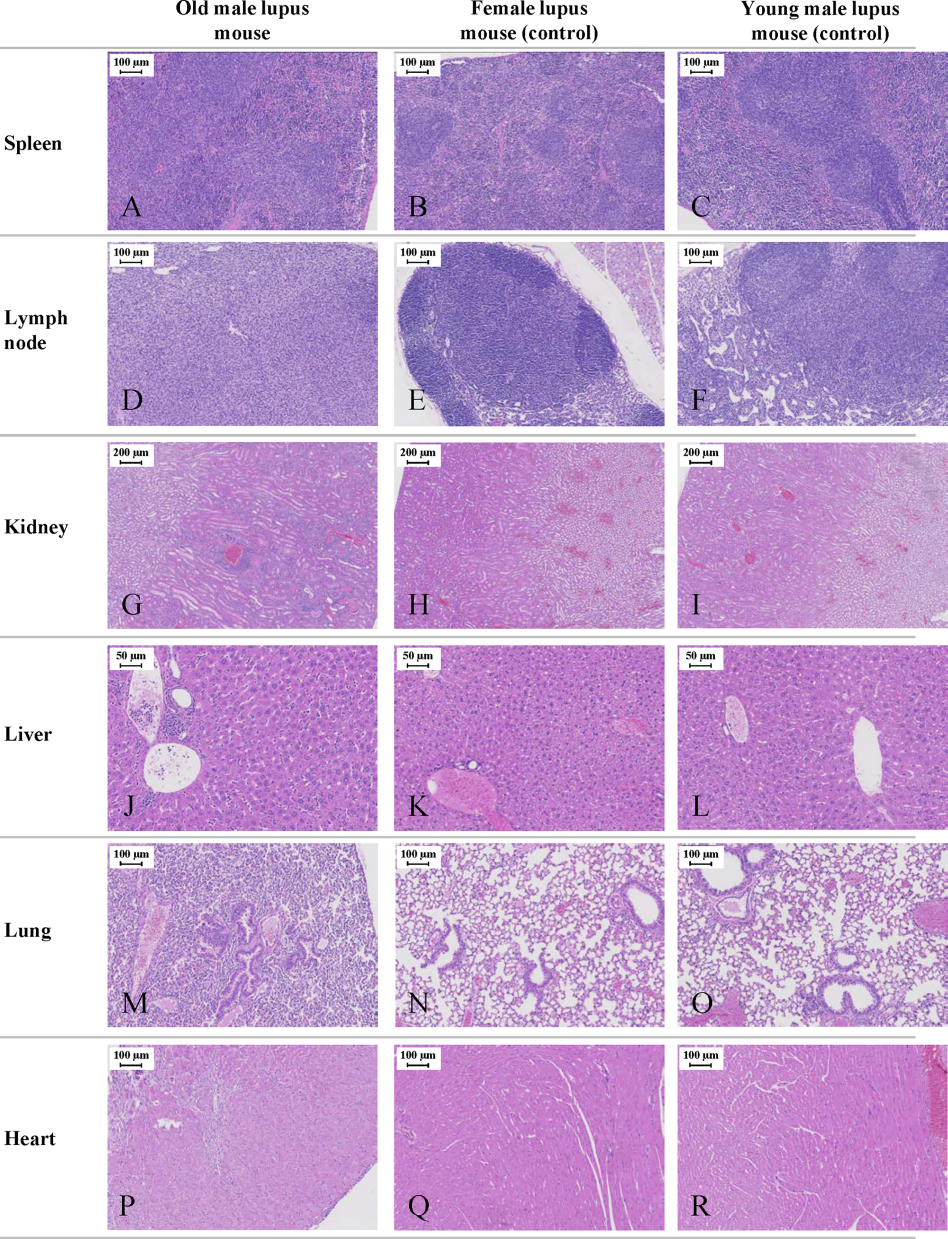
Representative histological images of the NZW/LacJ x BXSB/MpJ 9 F1 lupus mice. Internal organs (spleen, lymph nodes, kidneys, liver, lungs, and heart) were examined in 16-week-old male and female mice, and in young/healthy male mice (8 weeks). Young males without disease and female mice served as controls. Old lupus male mice showed abnormalities in multiple organs. Mild to moderate diffuse splenic lymphoid proliferation **(A)** compared to female **(B)** and young male lupus mice **(C)**. Marked lymphadenopathy with diffuse cortical and medullary lymphoid hyperplasia in multiple lymph nodes of old male **(D)** versus histologically normal lymph node architecture in female **(E)** and young male mice **(F)**. Moderate chronic multi-focal to segmental glomerulointerstitial nephritis **(G)** compared to unaffected female **(H)** and young male **(I)** mice. Moderate multifocal sinusoidal and periportal inflammatory cell infiltrates in the liver of old male animals **(J)**. Histologically normal liver from female **(K)** and young male **(L)** mice. Lung section from old male mice with pulmonary interstitial hypertrophy, intra-alveolar macrophages, and bronchiolar epithelial hyperplasia **(M)**. Histologically unremarkable lung sections from female **(N)** and young male mice **(O)**. Ventricular myocardium from old male mouse with moderate single cell to multifocal necrosis and mild to moderate multifocal myocarditis **(P)**. Normal ventricular myocardium in female **(Q)** and young male **(R)** mice. All sections stained with H&E.

Histological analysis was essential in our study to understand the impact of lupus on a variety of organs in this mouse model. The necropsy examination showed abdominal distension associated with serous effusion and histological examination identified multi-organ histopathology in old lupus male mice. These results were consistent with the physical/clinical observations in this study. Although no significant histopathology was identified in all organs examined in female and young male mice (spleen, lymph nodes, liver, kidney, lung, heart), old lupus male mice showed abnormalities in multiple organs (**Figure 5**). The spleens had marked splenomegaly (long axis average length 3.8 ± 0.5 cm) and histologically showed mild to moderate diffuse lymphoid proliferation (**Figure 5A**) compared to female and young male lupus mice (**Figures 5B, C**). Affected mice also had marked lymphadenopathy with axillary, mediastinal, lumbar, and popliteal lymph nodes enlarged up to 1cm in long axial length with diffuse cortical and medullary lymphoid hyperplasia (**Figures 5D–F**). Old male mice also had histopathological changes consistent with SLE’s autoimmune response including moderate chronic multi-focal to segmental glomerulointerstitial nephritis (**Figures 5G–I**). Congo red staining of kidney for amyloid was negative in all sections examined (*data not shown*). Liver sections of old mice were characterized by moderate multifocal sinusoidal and periportal inflammatory cell infiltrates (**Figures 5J–L**). The lungs from some old male mice were histologically unremarkable. However, in several old male lupus mice lung sections showed pulmonary interstitial hypertrophy, intra-alveolar macrophages, and bronchiolar epithelial hyperplasia (**Figure 5M**) compared to the histologically unremarkable lung sections from female and young male lupus mice (**Figures 5N, O**). In the mice with lung changes, abnormalities in the heart were also identified including moderate single cell to multifocal necrosis with mild to moderate multifocal myocarditis dominated by mononuclear cell infiltrates and myocardial fibrosis (**Figure 5P**).

## Discussion

The relationship between the progression of lupus disease and the physical/clinical changes in the (NZW×BXSB) F1 lupus-prone male mouse model has not been thoroughly studied or even considered. Although no single animal model can wholly represent all clinical symptoms in human patients with lupus, each model shares a particular subset of characteristics that mimic human SLE, making them valuable (4, 12, 13). In our study, we provide evidence that the lupus-like syndrome developed in NZW/LacJ x BXSB/MpJ (F1 hybrids) was progressive and fatal, where nearly 100% of the male mice developed severe clinical symptoms. While lupus can be challenging to diagnose due to the complexity and the heterogeneity of clinical signs and symptoms, it was even more difficult in this study because of the sudden unexpected death exhibited in this model, where more than 35% of the lupus male mice died between the age of 13-18 weeks. These findings indicated that the (NZW×BXSB) F1 lupus-prone male mouse model investigated in this study was associated with a more rapidly progressive and severe disease than previously reported using the same strain (14). Although the primary cause of death is still unknown, its occurrence positively correlates with the severity of lupus flare in this model.

The body condition and general activity of NZW×BXSB F1 as measures of animal well-being exhibited the first noticeable manifestation of the disease in this model, characterized by significant abdominal distension/swelling in the male mice as they age. In some cases, abdominal distension can be recognized even before observing an increase in the weight measurements. Swelling caused by lupus has also been described and reported in humans (15–18). While swelling may occur in humans across the whole body, it is usually recognized in the joints, ankles, feet, legs, neck and face/eyelids. Noteworthy, in this study, severe swelling was observed around the neck and face area in some of the old mice group, but no paw swelling was detected in our cases, suggesting that this model may not develop arthritis. Swelling associated with lupus often can be a sign of a more severe complication, especially since lupus can cause inflammation and tissue damage to any organ system in the body due to abnormal immunological function. The swelling observed in the old lupus male mice was consistent with our post-mortem necropsy findings, such as lymphadenopathy and kidney/renal impairment (lupus nephritis), resulting in diffuse edema and weight gain. It has been indicated that lupus nephritis is a common manifestation of SLE that can contribute to morbidity and mortality in SLE (19, 20). Symptoms may not be noticed in the early stages of lupus nephritis; however, as the inflammation continues to attack the kidneys, it prevents the normal kidney functions, leading to heavy loss of protein in the urine and fluid build-up in the body (edema). Lupus nephritis in our mouse model was also confirmed when urine collected from some of the old sick male mice with lupus showed a significant amount of protein leak in urine (2-3 plus; Urinalysis Test CHEMSTRIPS 9; **Supplementary Figure 3**).

As the disease gets worse in the old lupus male mice, it causes more substantial complications such as inability to walk and difficulties in executing movements, which were associated with the severity of other physical/clinical symptoms (e.g. rapid weight gain and swelling), age of male lupus mouse, and duration of illness. Additionally, we observed penile prolapse (40% of the old sick male mice) with inflammation (redness, swelling and bleeding sometimes; **Supplementary Figure 1**) in some cases. All the penile prolapse cases were associated with abdominal distension/swelling (moderate to severe), and most of these cases led to the inability to urinate or discharge normally and rarely survive after the diagnosis. Furthermore, the significant increase in the paleness of the paw pad with aging indicated poor blood circulation, which can be a sign of abnormal conditions such as hypoxia, anemia, or circulatory failure. This was reflected in the necropsy analysis (heart abnormalities in old lupus males) and the blood test which showed that the level of RBCs and hemoglobin were significantly lower in old lupus males compared to females and young males. Blood tests also revealed that the level of platelets was significantly lower in old males compared to females and young male mice, suggesting that these old sick male mice developed immune thrombocytopenia. We found anti-platelet and anti-RBC antibodies present in the sera from the old sick lupus mice (data not shown). The presence of anti-platelet antibodies was observed in 8 out of 12 (66.6%) sick mice, and 2 out of 10 (20%) sick mice showed anti-RBC antibodies in circulation. These results could partially explain the low RBC and platelet counts observed in circulation for the NZW x BXSB F1 old male lupus mice. While anti-RBC antibodies have never been investigated in this lupus mouse model, our findings of anti-platelet antibodies are in agreement with what was reported by Oyaizu et al. (21) in 1988, where (NZW/BXSB) F1 male mice were described as a new model for ITP. However, the ITP indications occurred slightly earlier in our study (within 4 months) compared to the reports by Oyaizu et al. (21)(> 5 months). It is worth noting that even though the 16-week-old female mice in this study showed no significant abnormalities, a previous study (14) showed that the NZW x BXSB F1 females develop SLE much slower than male mice. Whether the F1 female mice in our study may develop some of the SLE symptoms observed in our study or reach comparable levels as male mice later in age (e.g., 11 months of age or older) remains to be determined.

F1 hybrids of NZW x BXSB male mice showed similar course of SLE disease as that occurs in male BXSB parents, but more severe and progressive disease as they (F1 hybrids of NZW x BXSB male mice) fully express the Yaa gene of the BXSB strain (14, 22, 23). In our preliminary studies, the early disease onset (e.g., increased swelling, body weight, decreased activity, changes in blood cell parameters) is observed around 10 ± 2 weeks of age. Much more severe symptoms were developed with aging, and the most significant differences in the physical characteristics and clinical manifestations were detected around 15-16 weeks of age. Therefore, and because of the higher mortality rate after 16 weeks of age with this model, our experiments for this study were performed within 16 weeks. The physical changes, clinical manifestations, and the multiple organ damage observed in this lupus-prone male mouse model are more likely associated with the detected autoantibodies (e.g. anti-dsDNA antibodies) in the serum. The significant role of autoantibodies in the pathogenesis of lupus has been reported (24, 25), especially the anti-dsDNA antibodies, which are highly specific for lupus. It has been shown that the levels of the anti-dsDNA antibodies tend to reflect disease activity; therefore, repeated serology testing of these antibodies is common practice as it can help assess the diagnosis of lupus, prognosis, staging, and disease activity (26–29), even though not in all patients. While these pathogenic autoantibodies have been shown to be involved in the pathogenesis of lupus and their capabilities in affecting multi-organs and causing tissue damage (24, 25, 29, 30), they are not sufficient to cause the disease. Thus, since the exact mechanism by which lupus disease is developed remains to be clarified, additional studies are warranted to understand the actual cause and the multifaceted mechanisms underlying this complex disorder.

## Conclusion

Our goal of this study was to fill the gap in the literature by providing physical/clinical characterizations to evaluate the progress of the lupus disease and highlight the main challenges associated with the (NZW/LacJ x BXSB/MpJ) F1 lupus-prone mouse model. This study showed that NZW×BXSB F1 lupus-prone male mouse model is a complicated model that developed an early severe SLE-like disease. The physical and clinical examinations provided in this study revealed that the old lupus male mice exhibited many complex features of SLE, including the presence ofautoantibodies (e.g. anti-dsDNA antibodies), glomerulonephritis, lymphadenopathy, spleen and heart abnormalities in addition to the severe abdominal distension/swelling, inability to walk, and significant weight increase. Examining this mouse model was challenging due to the extreme progression development of the disease and the sudden unexpected death exhibited. Therefore, we hope that the characterizations and the knowledge provided in this study can be helpful to better evaluate and understand the progress of this disorder and may be used to assess the impact of different immunotherapies on ameliorating this complex disease.

## Data availability statement

The raw data supporting the conclusions of this article will be made available by the authors, without undue reservation.

## Ethics statement

The animal study was reviewed and approved by University Health Network (UHN) Animal Use Committee. Animal Use Permit AUP1788.26.

## Author contributions

DB conceived the study. DB, RA, and KFB contributed in the experimental design of the research. RA, KFB, and ML performed the experiments, contributed to data collection and analysis. RA and DB developed the outline and objective of this article. All authors contributed to the first draft of the article. RA took the lead on the project and in writing the manuscript. CM performed the histopathology analysis. DB, RA, KFB, and CM contributed to its editing. All authors contributed to the article and approved the submitted version.

## Funding

RA and KFB are supported by a Post-doctoral Fellowship from the Canadian Blood Services. ML is supported by a graduate scholarship from the Canadian Blood Services. Our research program received financial support from Canadian Blood Services, funded by the Federal (Health Canada), Provincial and Territorial Ministries of Health. The views expressed herein do not represent the views of the federal government.

## Acknowledgments

Authors would like to acknowledge Dr. Anna Pietraszek, Clinical veterinarian at ARC-UHN for necropsy and Charles River Research Animal Diagnostic Services for the histopathology services. Authors would like also to acknowledge Beth Binnington, senior research assistant at CBS/Innovation & Portfolio Management, for her support during the study.

## Conflict of interest

The authors declare that the research was conducted in the absence of any commercial or financial relationships that could be construed as a potential conflict of interest.

## Publisher’s note

All claims expressed in this article are solely those of the authors and do not necessarily represent those of their affiliated organizations, or those of the publisher, the editors and the reviewers. Any product that may be evaluated in this article, or claim that may be made by its manufacturer, is not guaranteed or endorsed by the publisher.
