# Supplementary material for: (NZW × BXSB) F1 male mice: An unusual, severe and fatal mouse model of lupus erythematosus
Source: Front Immunol. 2022 Sep 23;13:977698. doi: 10.3389/fimmu.2022.977698 (PMC9541624; doi:10.3389/fimmu.2022.977698)

Supplementary Material

Lupus manuscript
June 2022

Supplementary Figures

Supplement Figure 1: Abnormal physical changes observed with NZW/LacJ x BXSB/MpJ (F1) lupus-prone male mouse model along with aging.

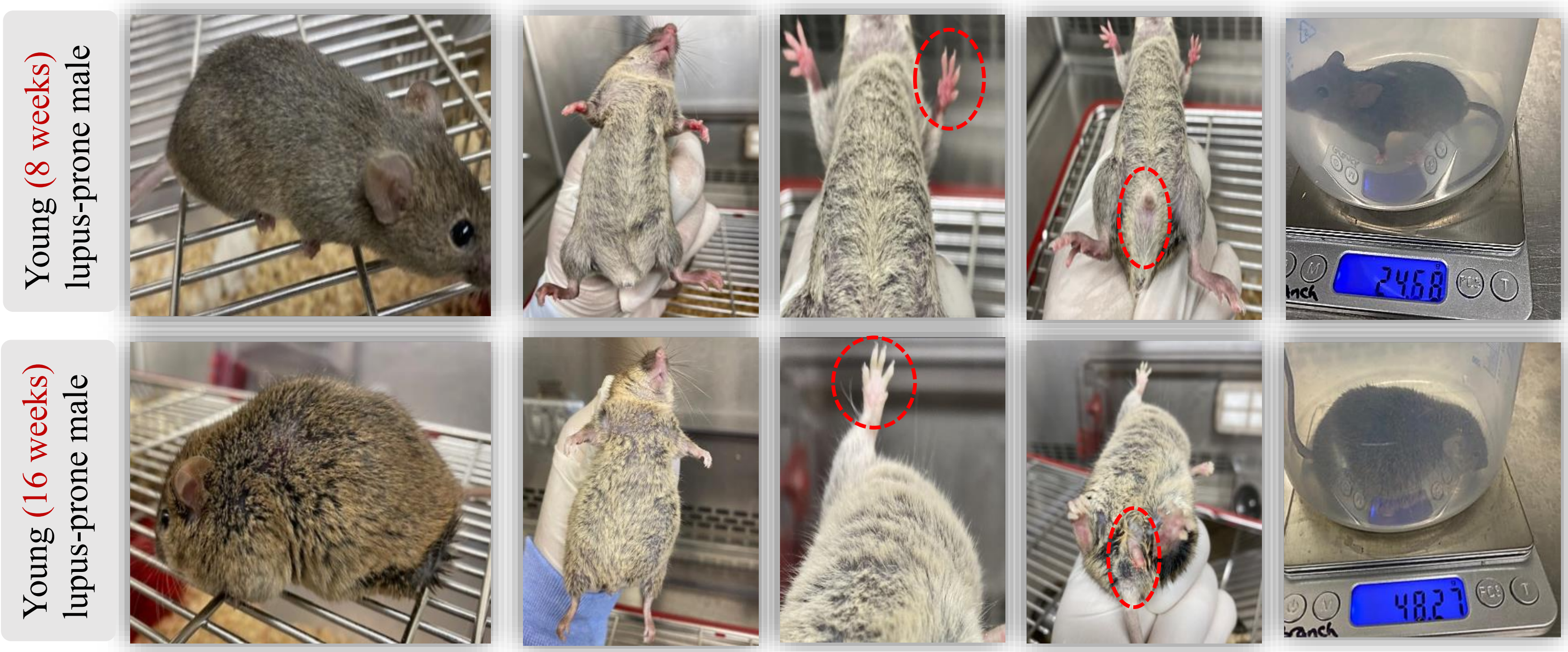

Supplements Figure 2: levels of anti-cardiolipin and– anti-nuclear antibody in female (green circle dots), young/healthy male (blue square) and old sick male (red tringle). Data are presented as scatter dot plot with median line. Results are expressed as Optical Density (OD) measurements using a microplate reader with a 450nm filter. P < 0.05 were considered statistically significant. Significant level (*p < 0.05; **p < 0.01; ***p <0.001, ****p < 0.0001) in comparison to controls.

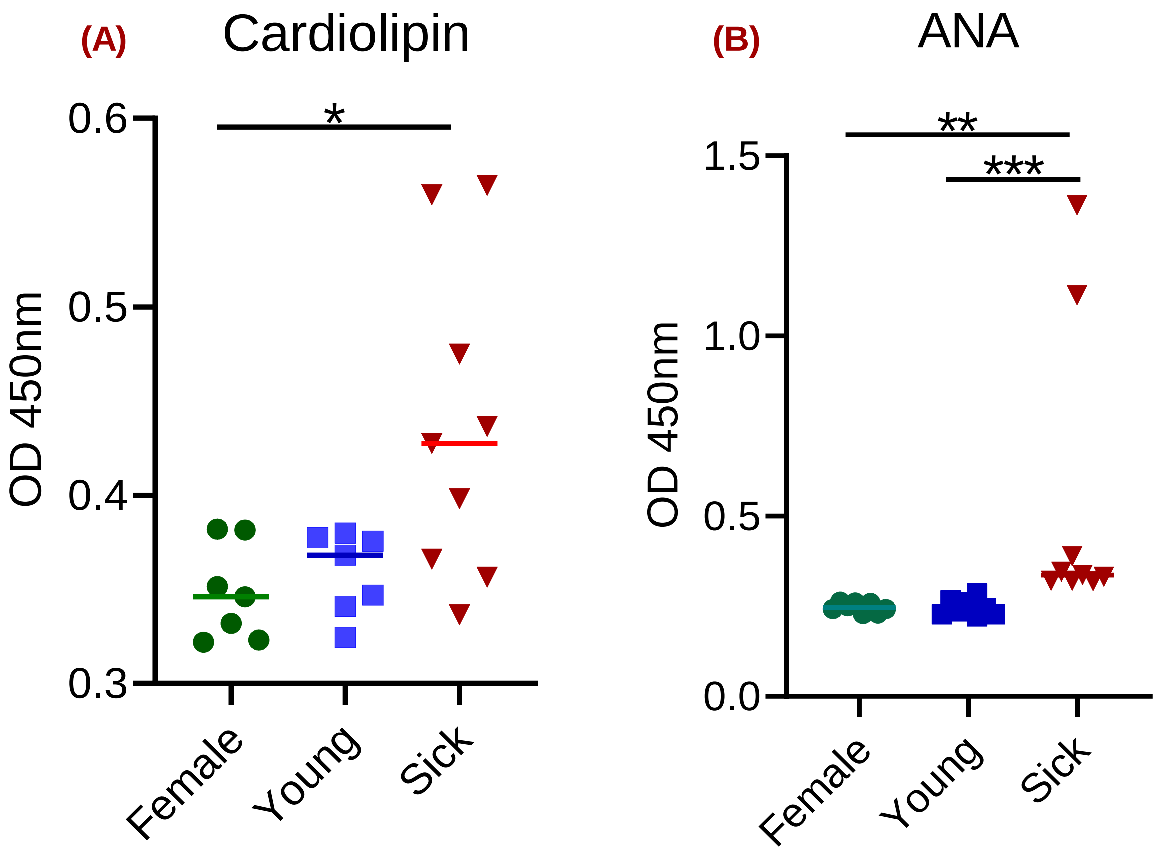

Supplements Figure 3: Urinalysis for NZW/LacJ x BXSB/MpJ (F1) old lupus male mice. The detection of nine of analytes in urine was completed using urine chemistry test strips (Roche Chemstrip 9 Urine Test Strips).

Urine Chem-Strips Results for Lupus Mice

Old Sick Mouse #1 (≥ 16 weeks)	Old Sick Mouse #2 (≥ 16 weeks)
Leukocytes (3+)	Leukocytes (2+)
Nitrite (negative)	Nitrite (negative)
pH (5)	pH (6)
Protein (3+)	Protein (2+)
Glucose normal	Glucose (normal)
Ketones (1+)	Ketones (negative)
Urobilinogen (normal)	Urobilinogen (normal)
Bilirubin (negative)	Bilirubin (negative)
Blood (2+)	Blood (4+)/ potential contaminant

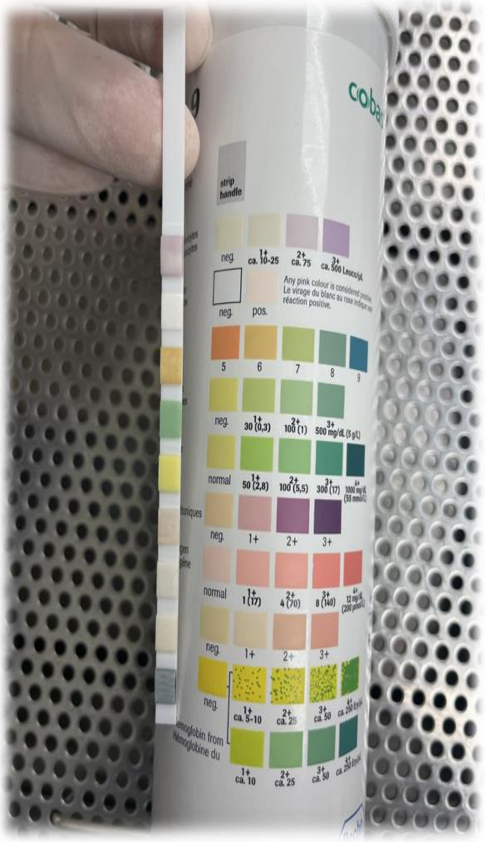

Supplement: Supplementary file 1 [file DataSheet_1.pdf]
